# An avirulent *Burkholderia pseudomallei* ∆*purM* strain with atypical type B LPS: expansion of the toolkit for biosafe studies of melioidosis

**DOI:** 10.1186/s12866-017-1040-4

**Published:** 2017-06-07

**Authors:** Michael H. Norris, Md Siddiqur Rahman Khan, Herbert P. Schweizer, Apichai Tuanyok

**Affiliations:** 1 Department of Infectious Diseases and Pathology, College of Veterinary Medicine, Univeristy of Florida, Gainesville, FL USA; 2 Department of Molecular Genetics and Microbiology, College of Medicine, University of Florida, Gainesville, FL USA; 3 Emerging Pathogens Institute, University of Florida, Gainesville, FL USA

## Abstract

**Background:**

The work was undertaken to expand the tools available for researching *Burkholderia pseudomallei* (*Bp*), the etiological agent of the tropical disease melioidosis. Melioidosis has the potential to pose a severe threat to public health and safety. In the United States, *Bp* is listed as a Tier-1 select agent by the Centers for Disease Control and Prevention (CDC), thus requiring high levels of regulation and biosafety level 3 (BSL3) facilities for experimental manipulation of live organisms. An avirulent ∆*purM* derivative of strain 1026b (Bp82) has proven to be a valuable tool for biosafe research as a select-agent excluded strain, but the high level of genetic diversity between *Bp* strains necessitates an expansion of the biosafe toolset.

**Results:**

The ∆*purM* mutation was recapitulated in the *Bp* 576a strain, a serotype B background. An important difference between strains 1026b and 576a is the lipopolysaccharide (LPS), a major virulence factor and protective antigen. Polyclonal sera from 1026b–challenged non-human primates showed no cross reactivity with strain 576a LPS and low reactivity with whole cell lysate. Strain 576a replicates to higher levels in mouse organs and induces more TNF-α in the lungs of BALB/c mice compared to 1026b. The newly created *Bp* 576a ∆*purM* strain, designated 576mn, was auxotrophic for adenine in minimal media, capable of wild-type growth in rich media with addition of adenine, and auxotrophy was abrogated with single-copy complementation. *Bp* 576mn was unable to replicate in human cells and was avirulent in BALB/c mice following high-dose intranasal inoculation, similar to Bp82. Organ loads indicated a significant reduction in bacterial replication.

**Conclusions:**

In this work, the new biosafe strain 576mn with atypical type B LPS was generated. This strain should prove a valuable addition to the toolkit for biosafe studies of *Bp* and development of therapeutic and preventative strategies aimed at combatting melioidosis. Strain 576mn is an ideal candidate for select-agent exclusion.

**Electronic supplementary material:**

The online version of this article (doi:10.1186/s12866-017-1040-4) contains supplementary material, which is available to authorized users.

## Background

*B. pseudomallei* (*Bp*), the etiological agent of the disease melioidosis, is a Gram-negative rod typically found in soil and water environments throughout the tropics [1]. In Thailand, numerous *Bp* are found in the pooled surface waters of rice paddies [2] coinciding with high disease and seropositivity rates in rural rice farmers. Compounding the problem is the quick progression to fatal sepsis; by the time patients seek medical intervention, the disease has progressed acutely, leading to a high mortality rate of 40.5% [3]. Melioidosis is not just a public health challenge in Northeastern Thailand. The disease is believed to be vastly underreported with ~165,000 cases worldwide and ~89,000 deaths [4]. Moreover, the US Centers for Disease Control and Prevention have listed *Bp* as a Tier-1 (top tier) select agent. Tier 1 organisms have the potential to pose a severe threat to US public health and safety and the US government agencies have invested resources to develop vaccines and therapeutics for them, including *Bp* [5].

Tier 1 listing dictates numerous regulations and requires select agent compliant biosafety level 3 (BSL3) facilities for manipulation of live organisms, driving up costs and increasing data collection time. One tool that can benefit the researchers in this restricted environment is the use of biosafe surrogates, which allow both exclusion from the select-agent regulations and safe manipulation at biosafety level 2 (BSL2). *Burkholderia thailandensis* (*Bt*) is an attenuated bacterium that is closely related to *Bp* but most strains lack a capsular polysaccharide, among other genomic and virulence factor differences, that do not make it an ideal biosafe surrogate [6]. *Bt* strain E555 has the capsular polysaccharide and has been successfully evaluated as a live-attenuated vaccine against *Bp* K96243 challenge [7]. There are currently three *Bp* strains that are excluded form the select agent list [8–10]. The first is an aminoglycoside pump mutant that lacks a capsule, JW270, another is a diaminopimelic acid (DAP) requiring Δ*asd* mutant, B0011, and the third is an adenine requiring Δ*purM* mutant, Bp82. Each individually has their drawbacks but cumulatively a major drawback is that all are made from the same strain background, *Bp* 1026b, a serotype A strain. As a species, *Bp* contains a high level of genomic diversity that translates to phenotypic diversity. The core genome of *Bp*, the genes found in all members of the species, is composed of 2570 genes [11]. Strain 1026b has 5782 genes. The available select-agent strains and *Bt* strains partially fulfill the need for surrogates in some aspects of study but there is room to expand the toolset.

Within-host, *Bp* can infect most tissues and invades, then replicates inside the cytoplasm of many cell types [8, 12–16]. To accomplish this feat, *Bp* attaches to the host cell causing actin rearrangement and inducing bacterial phagocytosis [17]. A myriad of virulence factors take part in the extra and intracellular lifestyle. Lipopolysaccharide (LPS) is the major component of the outer leaflet of the outer membrane and coats the surface of the Gram-negative bacterium, including *Bp*. Besides the ability of LPS to strongly activate innate immunity, it has been shown to play a role in the intracellular survival of *Bp* during invasion and mutants in the synthesis of the *O-*antigen of LPS are attenuated in animal infection models [18, 19]. A major variable among strains can be the lipopolysaccharide (LPS). Previously published research identified the genomic differences that exist at the *O-*antigen biosynthetic operon and screened ~1000 *Bp* strains for LPS diversity and 90% of them were type A (a.k.a. typical LPS) [20]. Strain 1026b has type A LPS. It has been found that the type A LPS *O-*antigen is composed of repeating subunits of glucose and talose [21]. Of the remaining 10% with atypical LPS; 9% were type B and 1% were type B2. Work presented in this study and in references cited above show a higher and larger banding pattern associated with the type B LPS. Observations by western blot show antibodies from patients infected with strains of one type are not cross-reactive. Lack of cross-reactivity between A and B serotype strains was assumed due to be differences in glycosyl residues of the *O*-antigen [20]. Recently, work from the authors has shown that the type B *O*-antigen is composed of rhamnose, xylose, and galactose (in a 4:1:1 M ratio, respectively) synchronizing the genomic and structural aspects of *Bp* LPS data [22]. Beyond Australia and Southeast Asia, the predominance of LPS types in South Asia, the Middle East, and Africa has been largely uncharacterized, but genomic data indicate a majority of strains identified in Madagascar have the type B LPS [23]. Even though the number of strains possessing atypical LPS is a fraction of the typical, their impact on the study of *Bp* and melioidosis is significant. Strain 576a was isolated from a fatal case of human melioidosis and has type B LPS [24, 25]. The most effective melioidosis vaccine thus far is 2D2, a branched chain amino acid auxotroph of *Bp* strain 576a containing a transposon insertion in the *ilvI* gene. It was an extremely effective vaccine in animal models, protecting BALB/c mice from a 10^6^ CFU challenge [25] whereas 1026b based vaccines fail to illicit long-term protection against 5 × 10^3^ CFU challenge doses [8]. The Melioidosis Vaccine Steering Committee has recommended that strain 2D2 be used as a positive control for vaccines in development [26]. Strain 2D2 is inaccessible to many researchers in the United States and is currently not a select-agent excluded strain. As mentioned above, this mutant is an insertional mutant, thus posing inherent risks from recombination proficient *Bp*.

We found significant differences between immunoreactivity of 1026b and 576a as well as organ loads and inflammation caused by these two wild-type strains in the BALB/c murine melioidosis model. The 1026b Δ*purM* strain, Bp82, has proven to be a valuable resource for studying antibiotic resistance mechanisms and vaccines [9, 27–30]. This work aims to expand on the availability, familiarity, and utility of Δ*purM* biosafe strains for research by producing the *Bp* 576a Δ*purM* strain that contains a 114 bp deletion in the *purM* gene. The newly created *Bp* 576a ∆*purM* strain, designated 576mn, was auxotrophic for adenine in minimal media, capable of wild-type growth in rich media with addition of adenine, and auxotrophy was partially abrogated with single-copy complementation. *Bp* 576mn was unable to replicate in human cells and was completely avirulent in BALB/c mice following high-dose intranasal inoculation, similar to Bp82. Organ loads indicated 576mn was unable to replicate in the organs tested. This strain should prove a valuable addition to the biosafe study of *Bp* and is an ideal candidate for select-agent exclusion and could serve as a safe background for creation of a live-attenuated double mutant vaccine strain.

## Results

### Strain background differences between *Bp* 1026b and 576a

**Fig. 1:**
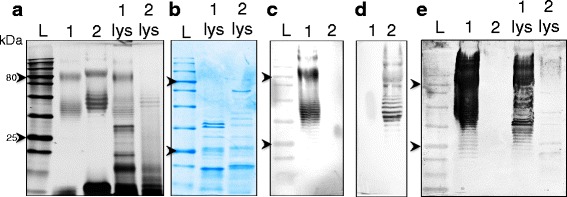
Strain background differences between *Bp* 1026b and 576a. **a** Silver stain of highly pure LPS from 1026b and 576a strain backgrounds and heat-killed lysate. **b** Coomassie stain of heat-killed cell lysate. **c** and **d** western blots using LPS type A specific mAb 4C7 and LPS type B specific mAb 5B4. **e** Western blot using day 28 serum from a rhesus macaque after aerosol challenge by *Bp* 1026b. Lanes: 1, FPLC-purified Bp82 Δ*wcb* LPS; 2, FPLC-purified 576a Δ*wcb* LPS; 1 lys, heat-killed lysate of Bp82; 2 lys, heat-killed lysate of 576mn. Chevrons indicate 80 kDa and 25 kDa as indicated

**Fig. 2:**
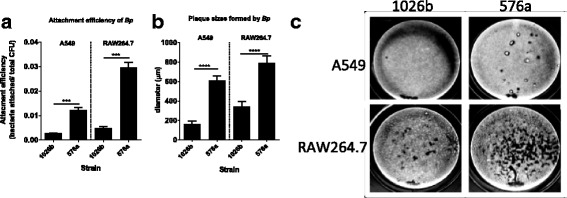
**a**-**b** Attachment and plaque sizes formed in A549 human lung epithelial cells and murine macrophage cell line RAW264.7 by *Bp* 1026b and 576a. **c** Images of plaque formation by 1026b and 576a in A549 and RAW264.7 cells after infection with the CFU. Error bars represent the SEM and differences found significant by one-way ANOVA

LPS purified from Bp82 and the 576a *wcbB* mutant show a fine laddering of *O-*antigen in the 1026b LPS on silver stained gels (Fig. 1a) but the type B LPS from 576a show a more pronounced laddering with a slightly higher molecular weight. Long and very-long LPS structures at ~80 kDa and beyond are also visible and of higher molecular weight in the 576a type B LPS. The lysates from Bp82 and 576mn show a conservation of these high molecular weight LPS structures. The Coomassie stained SDS-PAGE gel shows that protein production by these two strains shows some similarities but also differences (Fig. 1b). Western blots of the FPLC-purified LPS from 1026b and 576a with mAbs to the respective LPS types showed no cross-reactivity between the two strains (Fig. 1c-d). A Western blot using serum from a rhesus macaque 28 days after aerosol challenge with *Bp* 1026b showed that the serum reacted strongly to the FPLC - purified type A LPS from 1026b and not at all to type B LPS from 576a (Fig. 1e, lanes 1 and 2, respectively). The highly immunogenic potential of *Bp* LPS during the humoral response is evident in the strength of macaque serum reactivity to Bp82 lysate compared to reactivity with 576mn cell lysate (Fig. 1e, lanes 1 lys and 2 lys). There is faint reactivity to some proteins from 576mn but not to the major antigen LPS. The identity of these proteins will be determined in future work. Numerous serum samples from rhesus macaques were used in an ELISA using purified LPS from 1026b and 576a capsule mutants in two separate pure antigen assays. All monkeys were challenged with *Bp* strains that have type A LPS. The reactivity of the serum with the type A LPS increases starting at 7 days post-infection and the mean OD_450_ remained high in the serum samples of surviving monkeys (Additional file 1). In contrast, there was very little reactivity between the type B LPS and all serum samples compared to serum from mice vaccinated with type B LPS, agreeing with the blot in Fig. 1e. It was also found that in human lung epithelial A549 cells and mouse macrophage RAW264.7 cells that *Bp* 576a attached better and formed larger and more numerous plaques (Fig. 2a-c), indicating further differences between these strains at the cellular infection level.

### *Bp* 1026b and 576a performance in a murine melioidosis model

**Fig. 3:**
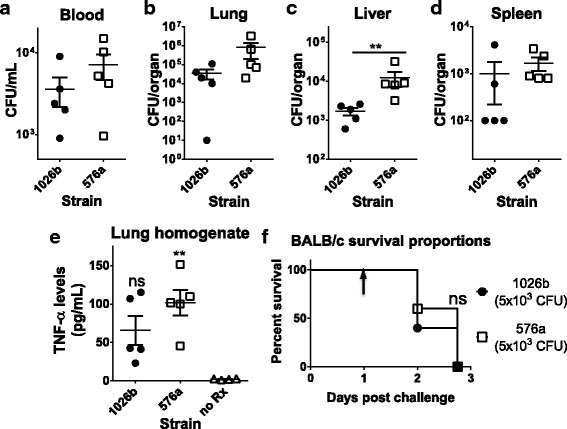
Bacterial burdens and TNF-α levels are different in the organs of BALB/c mice infected with *Bp* 1026b or *Bp* 576a. **a** Bacterial CFU/ml of blood and bacterial CFU/organ, i.e. (**b**), Lung; (**c**), Liver; (**d**), Spleen. Statistical significance determined by Mann-Whitney statistical testing of the medians of the two groups. ** = *p* < 0.01 E), TNF-α levels in lung homogenate from 1026b are not significantly (ns) different than uninfected mice while TNF-α levels from 576a treated lungs were significantly different than untreated mice lungs by the One-way ANOVA test for comparing the three groups. ** = *p* < 0.01. Black circles indicate 1026b–infected mice. White squares indicate 576a–infected mice. White triangles indicate uninfected mice. **f** BALB/c survival proportions following intranasal challenge with 5000 CFU of each strain indicated. The black arrow indicates the 24 h time point for removal of organs during the pre-determined endpoint study in (**a**-**e**), the survival data is also presented in Fig. 7 for clarity. ns = not significant by the Log-rank test of survival curves

Using the BALB/c murine model of infection we also compared the organ dissemination characteristics of the parental wild-type *Bp* strains 1026b and 576a. Mice challenged with 5000 CFU of either strain were sacrificed at 24 h post-infection and had the bacterial organ loads in blood, lung, liver, and spleen enumerated (Fig. 3a-d, respectively). The data show that mean CFUs of 576a bacteria in the blood, lung, and spleen trended higher but that the levels were not significantly different. Mean 576a bacterial levels in the liver were significantly higher. Ten times more CFUs were recovered from 576a–infected mouse livers compared to livers from 1026b–infected mice and this difference was highly significant (Fig. 3c). This may be due to the increased immune activation of 576a LPS [31] and activation of TLR4-dependent bacterial clearance and uptake by the liver during sepsis [32]. Although bacterial burden in the lung was not significantly different between 1026b and 576a infected mice, TNF-α levels were measured to characterize the acute-phase inflammatory responses to bacterial replication in the lung (Fig. 3e). TNF-α levels in lung homogenate from 1026b–infected mice trended higher but were not significantly different than in uninfected mouse lungs. TNF-α levels in lung homogenate from 576a infected mouse lung homogenates were significantly higher than those from the uninfected mice but not from 1026b infected mice. This could be due to slightly higher levels of bacteria in the lung or to the higher immunogenicity associated with this strain. Regardless, in mouse survival curves following intranasal challenge with 5000 CFU of either wild-type strain indicate all mice are moribund prior to 3 days post-infection and survival of mouse groups infected by the two strains are not significantly different (Fig. 3f; and also presented in Fig. 7 for clarity).

### Construction and growth characterization of a 576a Δ*purM* mutant

**Fig. 4:**
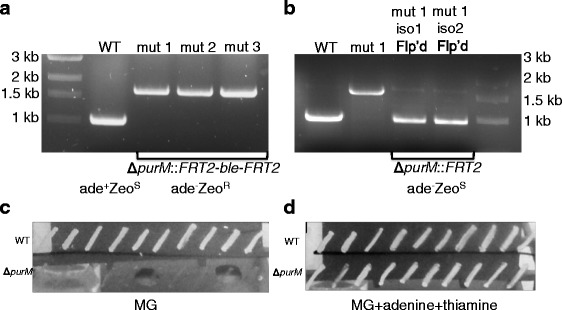
PCR verification of *Bp* 576a ∆*purM*::*FRT2-ble-FRT2*, *ble* selection marker removal, and adenine auxotrophy screening. **a** Agarose gel showing the PCR product from the wild-type (WT) 576a *purM* region and the shift-up after deletion by insertion of the *FRT2-ble-FRT2* cassette in three mutants (mut1–3). **b** Agarose gel showing the PCR product from the wild-type (WT) 576a *purM* region, the shift to a larger size after deletion of an internal *purM* fragment and insertion of the *FRT2-ble-FRT2* cassette and the shift to a smaller size after Flp-excision of the *ble* cassette in two isolates. Genotypes and phenotypes are indicated below panels (**a**) and (**b**). Patch plates showing the phenotype of 576a wild-type (WT) and 576mn patched on minimal glucose (MG) + thiamine media (**c**) and on MG + adenine + thiamine media (**d**)

**Fig. 5:**
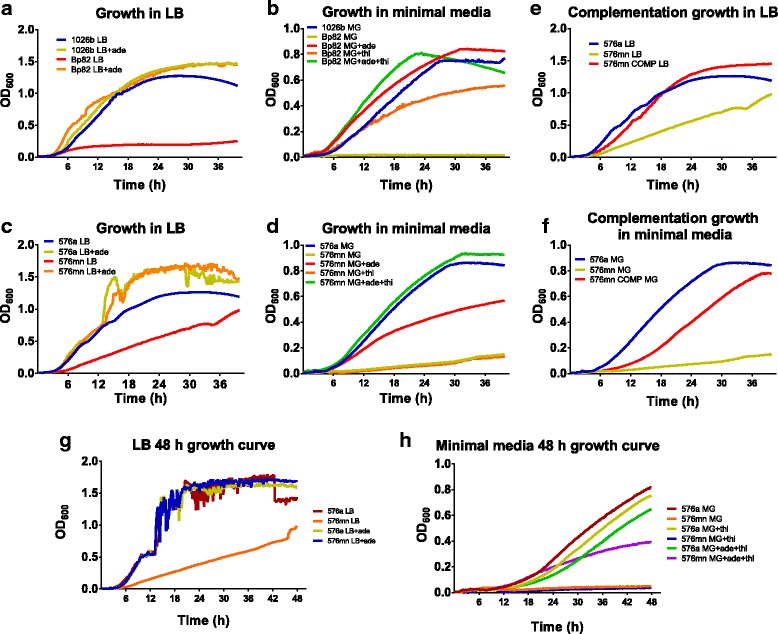
Growth analysis of *Bp* 576mn in rich and minimal media. Growth phenotypes of *Bp* strains 1026b and Bp82 were carried out as controls after 1:200 dilutions in LB (**a**) and minimal glucose media (**b**) +/− adenine and thiamine supplements as indicated. Growth phenotypes of *Bp* strains 576a and 576mn in LB (**c**) and minimal glucose media (**d**) +/− adenine and thiamine supplements as indicated. Growth curves of the 576mn COMP compared to 576a and 576mn in LB (**e**) and minimal glucose media (**f**) showing full complementation in LB but partial complementation in minimal glucose media. Growth phenotypes of 576a and 576mn in rich media (**g**) or minimal media (**h**) after 1:500 dilutions out to 48 h +/− adenine and thiamine as indicated

Allelic recombination was utilized to delete a 125 bp fragment internal to the *purM* gene by insertion of the *FRT2-ble-FRT2* marker and selection on Zeocin. Insertion of the marker was verified and three isolates were screened by PCR and patching as described in Methods. The presence of the mutant allele was first characterized by PCR. Replacement of the internally deleted *purM* DNA sequences with the *FRT2-ble-FRT2* fragment resulted in a shift in PCR product size from 1 to 1.6 kb (Fig. 4a). This mutant genotype was accompanied with the correct adenine auxotrophy and zeocin resistance phenotypes. After Flp-mediated selection marker excision and curing of the Flp expression plasmid, the size of the PCR product obtained with the same primer set from the resulting zeocin susceptible adenine auxotrophs was reduced to 930 bp (Fig. 4b). As expected, cells of *Bp* 576a Δ*purM*::*FRT2-ble-FRT2* did not grow on minimal glucose media unless supplemented with adenine and thiamine (Fig. 4a, c and d). The observed growth phenotypes and PCR product patterns were consistent with those previously observed for Bp82 [9]. Growth analysis by growth curve was carried out using strains 1026b and Bp82 as positive controls (Fig. 5a and b). Without adenine Bp82 was unable to replicate well in rich LB media. Addition of adenine to the media restored wild-type growth to Bp82 (Fig. 5a). In minimal media, thiamine partially restored growth. Supplementation with adenine alone afforded Bp82 growth similar to wild-type 1026b and both adenine and thiamine increased the growth rate a little further. In rich LB media the markerless *Bp* 576a Δ*purM*::*FRT2* strain, henceforth called 576mn, required adenine supplementation to grow the same as wild-type 576a. Without adenine the growth rate was halved (Fig. 5c). In minimal media *Bp* 576mn required both adenine and thiamine for restoration of wild-type growth rates. Thiamine alone was not sufficient while adenine alone only partly complemented (Fig. 5d). *Bp* 576mn was complemented using a mini-Tn*7* vector with a wild-type *Bp purM* gene. In rich media the complementation was complete and 576mn COMP was able to grow the same as wild-type 576a (Fig. 5e). The single-copy complementation was incomplete in minimal glucose media but did allow the growth at half the rate of wild-type 576a without supplementation (Fig. 5f). By increasing the primary dilution factor to 1:500 and time of study we sought to ensure the strain was unable to grow in minimal media without adenine and thiamine supplementation. In Fig. 4g and e, show that strain 576mn grows poorly in LB without adenine and is unable to grow after 48 h in minimal glucose media without adenine and thiamine supplementation. These data show that 576mn is an adenine auxotroph.

### Intracellular replication of Δ*purM* mutants in the human cell-line HEK293.

**Fig. 6:**
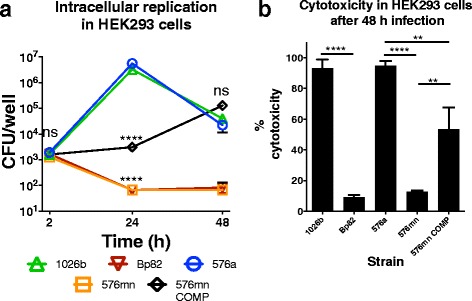
*Bp* 576mn is unable to replicate in the human cell line HEK293 intracellular replication model and is not significantly cytotoxic. **a** Aminoglycoside protection assay revealed that *Bp* 576mn (orange square) was unable to replicate inside HEK293 cells compared to 576a WT (blue square). Strain 1026b (green triangle) and Bp82 (maroon triangle) were included as controls. Strain 576mn COMP was only partially returned to wild-type levels of replication (black diamonds). The symbols are the average of experimental data acquired in biological triplicate. The SEM is not visible at the scale presented. The differences between all strains at 2 h were not significant. Significance determined by Two-way ANOVA. ns = not significant, ** = *p* < *0.01* **** = *p < 0.0001.* **b** Cytotoxicity of HEK293 cells after 48 h infection with the indicated strains. Significance determined by one-way ANOVA. ns = not significant, ** = *p* < *0.01* **** = *p < 0.0001*

In a crucial step in demonstrating the biosafe utility of this strain, we wanted to ensure that the new *Bp* 576mn strain was unable to grow in human cells. HEK293, a human embryonic kidney cell line used for *Bp* intracellular infection experiments [33–35], was used to determine if 576mn was capable of invasion and intracellular replication in human cells (Fig. 6a). All strains showed no significant difference in invasion efficiency (Additional file 2). Demonstration of Bp82 replicative ability in human cells was never investigated so we included Bp82 in the HEK293 infection model and compared it to wild-type 1026b. Bp82 invaded the same as 1026b but showed over a log reduction in intracellular replication at 24 h post-infection that remained on the verge of undetectable for the 48 h time point. Strain 576a replicated similarly to 1026b but with a slightly higher CFU at 24 h. Previous experiments have shown that total lysis of monolayers by intracellular *Bp* can occur after 24 h of infection. The drop in intracellular wild-type CFU between 24 and 48 h is indicative of lysis of host-cells. Strain 576mn invaded the HEK293 cells at a similar efficiency as the wild-type bacteria and intracellular CFU levels among all strains were not significantly different at 2 h (Fig. 6a). Intracellular CFU of 576mn at 24 h were decreased in comparison to the 2 h time point and mirrored the log reduction in levels from the 2 to 24 h seen with Bp82. Differences in CFU of wild-type 576a and 576mn at 24 h were significant. In comparing the complemented 576mn strain, 576mn COMP, the complementation allowed some intracellular replication but there was still 1000 times less bacteria than wild-type at 24 h. The single chromosomal copy of *purM* was only able to partially complement the defect in intracellular replication of the 576mn mutant. The intracellular CFU of the 576mn complement at 24 h slightly increased in relation to the 2 h time point and by 48 h showed a 2-log increase in intracellular CFU. Data from the growth curve indicated a reduction in growth rate of 576mn COMP in rich media that possibly translated to inefficient growth within HEK293 cells.

Cytotoxicity in HEK293 cells was also measured at the 48 h time point (Fig. 6b). HEK293 cells infected with either wild-type 1026b or 576a exhibited almost 100% cytotoxicity. In comparison, both *purM* mutants had levels hovering around 10%, significantly lower than their respective wild-type strains. The complemented 576mn strain caused ~50% cytotoxicity that was in between the wild-type and *purM* mutants.

### Strain attenuation testing in the murine melioidosis model

**Fig. 7:**
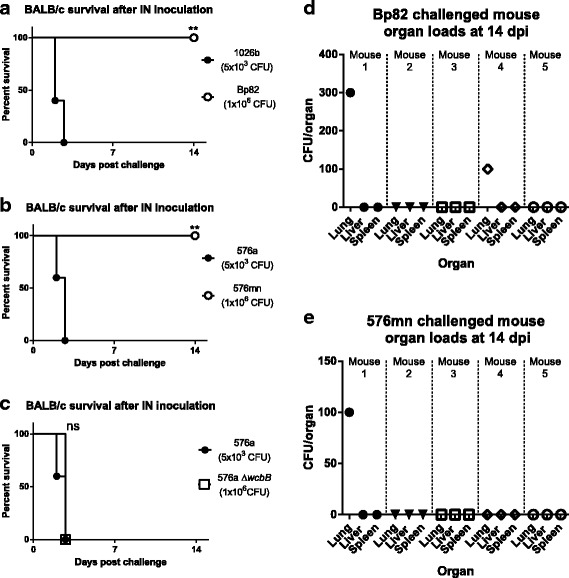
*Bp* strain 576mn is attenuated in the BALB/c mouse model of melioidosis and is efficiently cleared from the organs. **a** BALB/c mice challenged intranasally with 5 × 10^3^ CFU of wild-type *Bp* 1026b succumbed to infection within 3 days while mice intranasally challenged with 1 × 10^6^ CFU of Bp82 survived until the end of the study. **b** BALB/c mice intranasally challenged with 5 × 10^3^ CFU of wild-type *Bp* 576a succumbed to infection within 3 days while mice challenged intranasally with 1 × 10^6^ CFU of 576mn survived until the end of the study. The increased survival by mice challenged with the mutant strains was highly significant in (**a**) and (**b**). **c** A 1 × 10^6^ CFU intranasal challenge of the *Bp* 576a Δ*wcbB* mutant exhibited the same lethality as wild-type *Bp* 576a. **d** Organ loads from Bp82 challenged mice at the end of the 14 day study show 2/5 mice have detectable numbers of bacteria in their lungs. **e** Organ loads from 576mn challenged mice at the end of the 14 day study show 1/5 mice have detectable numbers of bacteria in their lungs, a 4-log reduction in bacterial numbers. Significance was determined by the Log-rank Mantel-Cox test. ns = not significant, ** = *p* < 0.01

Most importantly, the new adenine auxotrophic strain, 576mn, was tested for attenuation in mice. A control experiment was first carried out where 1026b and Bp82 were compared in the BALB/c inhalation mouse model. 5000 CFU (LD_50_ ~ 900 CFU [36]) of wild-type 1026b and 1 × 10^6^ CFU of Bp82 were intranasally inoculated into the nares of anesthetized mice and observed for moribundity (Fig. 7a). Mice challenged with ~5 times the LD_50_ of wild-type 1026b (5000 CFU) became moribund 2–3 days post-infection. Mice challenged with 1 × 10^6^ CFU Bp82 (~1000 times the LD_50_ of 1026b) appeared healthy until the study endpoint, 14 days post-infection. Substantiation of the attenuation of Bp82 in the BALB/c intranasal model led us to testing 576mn in the same manner (Fig. 7b). Mice infected with 5000 CFU wild-type *Bp* 576a succumbed quickly to infection. Similar to Bp82, mice challenged with 1 × 10^6^ CFU of 576mn (~1000 times the LD_50_) showed no sign of illness and survived until the study endpoint, 14 days post-infection. As a comparison the capsule mutant 576a Δ*wcbB* was used to infect BALB/c mice intranasally at the same dose as 576mn (1 × 10^6^ CFU) (Fig. 7c). At the dose used, the 576a Δ*wcbB* capsule mutant was just as virulent as wild-type. At the end of the study, organs from the Bp82 and 576mn mice were removed and the bacterial organ loads were determined (Fig. 7d-e). Bacteria were not found in the spleens or livers of any of the mice. Two mice challenged with Bp82 had low amounts of bacteria still present in the lungs following high dose lethal challenge (Fig. 7d). The 300 and 100 CFU counts in two of the mice represent an average ~ 7000 times decrease in bacterial load compared to the 1 × 10^6^ CFU inoculum, indicating 3 of the mice completely cleared the initial challenge dose. Lungs of one mouse from the 576mn challenged group had 100 CFU bacteria present at the end of the study, representing a 10,000 times decrease in CFU compared to the inoculum CFU. The presence of 576mn bacteria in one mouse at low levels and the absence of bacteria in 4 out of 5 mice suggests it was more efficiently cleared than the Bp82 strain.

## Discussion

The *Bp* type strain 1026b has dominated vaccine studies, antibiotic resistance mechanism studies, virulence related studies and many others. The accessibility of the biosafe 1026b derivative strain Bp82 has been a boon to such studies by reducing the need for BSL3 manipulation and exclusion from select agent regulations. It has also proven to be an ideal source of material for immunological and vaccinological studies. Major differences in antigenic and genetic properties exist amongst *Bp* strains. Another type strain, 576a, has been used for numerous studies by groups in the UK. These two strains have many genetic differences, the most notable of which is the *O-*antigen structure. Antibodies raised against the LPS (type A) appeared to be a major component of the humoral response and of the three measured (antibodies to type I and II OPS, and flagellin) elevated levels of anti-type II OPS (LPS) antibodies were found to be the only antibody correlated with patient survival [37]. LPS subunit vaccines were also found to produce high levels of IgM and IgG and protected 60% of mice from IP challenge to day 35 but not from aerosol challenge [38]. So, it stands to reason that LPS structure impacts vaccine design and efficacy. Existence of a biosafe strain from the 576a background would allow for an increased ability to produce an array of immunogenic material from *Bp* and increase the biosafe toolkit available to investigators working under time, regulatory and cost limitations.

The data show that both LPS and protein content are different between the two strains. We demonstrated significant differences in immunogenicity using non-human primate serum and performance between 1026b and 576a in the animal model. We have also shown that the LPS of 576a is more immunogenic than 1026b [31] that may lead to increased virulence of this strain. We consistently found the proportion of acylation (increased) and degree of hydroxylation (decreased) were different in the lipid A of 576a compared to 1026b. In other work in preparation we found that in response to increased growth temperature, strain 1026b begins to modify the lipid A structures while 576a does not. Many intracellular pathogens reduce the immunogenicity of the lipid A portion of LPS to avoid activating the immune response in this manner [39–42]. While there may be some similarities in the pathogenesis of tularemia, plague, and melioidosis, *Yersinia pestis* and *Francisella tularensis* require a host for replication. Wild-type *Bp* can grow freely in diverse environments. So replication in the host is not an essential part of the lifecycle but more of an accidental occurrence and modification of the lipid A may be an adaptation to the host environment. Our hypothesis is that some *Bp* strains receive selective pressure while in the host and attempt to decrease immune recognition. Others do not and the increased stress response enhances production of virulence factors. This requires more work in the future, but the modifications are strain specific and most likely depend on the natural history of each strain. 1026b was isolated form a non-fatal case of septicemic melioidosis and has an LD_50_ by intraperitoneal injection in BALB/c mice of 5.1 × 10^4^ CFU [43]. Strain 576a was isolated from a fatal disseminated melioidosis case and has an LD_50_ of 80 CFU in the same murine melioidosis model [25]. In our BALB/c intranasal challenge model, 5000 CFU of either strain was used in a pre-determined endpoint study and showed that 576a induced higher levels of inflammation, by measure of TNF-α, in the lungs of infected mice than 1026b when compared to the PBS control. For consistency, 5000 CFU was also used to produce an acute infection and survival in the two groups of mice was not significantly different.

The decision to recapitulate the ∆*purM* mutation specifically in the *Bp* 576a strain background was based on the following criteria: 1. Bp82 (the 1026b ∆*purM* mutant) is a popular widely utilized strain and a whole cell vaccine based on the strain is effective [27, 44–48]. This work sought to build on the familiarity and accessibility of Bp82. 2. Strain 576a has LPS *O-*antigen that is different than the other exempt strains. 3. Besides all being of the same strain background, the other select agent exempt strains have the following drawbacks. Strain B0011 is a Δ*asd* mutant but the growth rate is affected in rich media compared to wild-type, while the capsule mutant, JW270, may still be virulent at the same challenge doses as seen in our capsule mutant strain made in this study. The capsule is a major virulence factor and immunogenic polysaccharide that would be important in the utility of a biosafe strain. 4. The previously published success of a vaccine based on strain 576a made this a desirable strain background for isolation of immunologic material and for comparison to the large amount of published data using strain 576a. 5. The aforementioned 2D2 strain is not excluded from select agent regulations, difficult to obtain, and is an insertion mutant that poses a risk of genetic reversion, thus necessitating the present work. We propose that any live attenuated vaccine strain based on 576a with actual potential should include a combination of *purM* and *ilvI* mutations.

Successful creation of the Δ*purM* mutation in the 576a strain background was verified by a strict requirement for adenine in minimal media by the patching of colonies. The effect of the Δ*purM* mutation on the growth of 576a was modest in rich media and could be overcome by the addition of adenine to LB media. The ability of thiamine to overcome the *purM* mutation is variable and may be due to small amounts of adenine crossover during growth preparation or growth stage of the bacteria used for inoculum dilution. Growth lag by the complement in minimal glucose media is more than likely due to the absence of important upstream regulatory elements not cloned into the single-copy complementation vector. Even though growth is delayed, the final cell density is equal to the wild-type. The phenotype of 576mn is consistent with Bp82, as previously published and in our hands. Further extension of growth curves to 48 h showed there was no measureable growth in minimal media without adenine and thiamine supplementation. Each of the two Δ*purM* strains was unable to grow within the cytosol of HEK293 cells. After 10^3^ cells invading, intracellular CFU of both strains decreased by a log and a half and hovered near our limit of detection out to 48 h. They were able to invade the cytosol but failed to replicate. The wild-type strains both had an ~10^3^ increase in intracellular CFU by 24 h post-infection while it took the 576mn COMP 48 h to reach ~10^5^ CFU. Cytotoxicity measurements showed the wild-type strains (~100%) and COMP (~50%) afforded significantly higher levels of cellular damage upon the HEK293 cells compared to Bp82 or 576mn (~10%). The relatively mild cytotoxic effect of the *purM* mutants agrees with the intracellular CFU data.

We demonstrated that Bp82 and 576mn are fully attenuated in the intranasal BALB/c mouse model even when inoculated with 200 times more CFU than wild-type. The 576a Δ*wcbB* mutant was also tested in the same approach. The difference in mouse survival between the mice challenged with 5000 CFU wild-type 576a and 1 × 10^6^ CFU of the 576a Δ*wcbB* mutant was not significantly different with all mice becoming moribund by 3 days post-infection. Even without a capsule, *Bp* 576a can be virulent at higher doses, similar to the select agent excluded strain JW270 and *B. thailandensis* [10]. These survival experiments indicate that 576mn behaves the same as Bp82 and that it is more attenuated than a Δ*wcbB* capsule mutant in the same background. One of five mice contained detectable bacteria in the lung after 14 days infection with a massive dose of 576mn, indicating the mutant is steadily cleared and would possibly be completely absent in a longer-term study. The absence of any detectable CFU in livers and spleens of any animals challenged with either Δ*purM* mutant indicate inability to disseminate and establish infections at secondary sites.

## Conclusion

It has been demonstrated that 1026b and 576a are very different strains of *Bp* especially in terms of the LPS *O*-antigen and total cellular protein patterns. Besides being a major virulence factor and a protective antigen, LPS is a highly immunogenic potentiator of immune responses. At the doses tested, strains 1026b and 576a are no different in their lethality in the BALB/c mouse model of melioidosis. Select-agent excluded strains have been crucial to the development of vaccines, particularly as sources of immunogenic material, such as outer membrane vesicles. Available select agent excluded *Bp* are all based on the same 1026b strain background, limiting the tools available for studying a highly diverse species. We are aiming to fill that gap by engineering and testing the 576a Δ*purM*::*FRT2* mutant, 576mn. This work demonstrated the auxotrophy of the mutant and its ability to grow in rich media, while being unable to replicate in minimal media after sufficient dilution or within human cells compared to the parental wild-type *Bp* strains. Strain 576mn is unable to significantly damage human cells and is avirulent in mice. It exhibits higher attenuation than the 576a Δ*wcbB* capsule mutant in the BALB/c mouse model. Strain JW270, which is 1026b with the acapsular phenotype, has already been excluded from select agent regulations even though it was as virulent as the 576a acapsular mutant tested in this work. Of the two strains evaluated, strain 576mn is the superior candidate for select-agent exclusion and will build on the utility and success of strain Bp82.

## Methods

### Bacterial strains and culture conditions

All Select Agent work was carried out in a CDC/USDA Tier 1 approved facility at the University of Florida following Tier 1 regulations. All protocols were approved by the Institutional Biosafety Committee prior to implementation. *Bp* strains (CDC/USDA registered in house bacterial inventory) were grown on Lennox broth (5 g/L NaCl) (LB, Fisher BioReagents) or Tryptic Soy Agar (Becton Dickinson) and grown at 37 °C. LB broth was used for liquid growth of all strains. LB supplemented with 1000 μg/mL kanamycin (Km, Fisher Scientific) and 2000 μg/mL zeocin (Zeo, Invivogen) was used for selection of mutants in *Bp* strains. Blue-white selection of pExKm5 derivatives using 5-bromo-4-chloro-3-indolyl-β-D-glucuronic acid (X-gluc) and counter-selection using 15% sucrose were accomplished as previously published [32, 49, 50]. *E. coli* strain NEB5α was used as a cloning strain (New England Biolabs). Selection of Km resistant *E. coli* strains was performed on LB medium with 35 μg/mL Km. Select agent excluded strain Bp82 [9] was grown on LB or TSA with 0.6 mM adenine (Amresco). Media for auxotrophy testing was M9 minimal salts with 20 mM glucose +/− 0.0005% thiamine +/− 0.6 mM adenine. Growth curves were carried out by diluting cultures 1:200 in media and shaking cultures in a 96-well flat bottomed plate with lid at 425 rpm in a BioTek Synergy HTX plate reader in duplicate at 37 °C. Forty eight hour studies were carried out by initial dilutions at 1:500. The optical density at 600 nm was measured every 10 min. The 576a Δ*wcb* capsule mutant was created by the authors and is described elsewhere (Norris et al., submitted). Human cell lines HEK293 and A549 and murine cell line RAW264.7 (American Type Culture Collection, ATCC) were grown in Dulbecco’s Modified Eagle Medium (DMEM) - high glucose + L-glutamine (HyClone) with 10% FBS (HyClone) in 5% CO_2_ at 37 °C. All plastic ware was Corningware with CellBIND surface. Culturing cells was carried out essentially as described previously [8, 35, 51, 52]. BD dye free Matrigel at 1:40 dilution in PBS was used to coat plates for 30 min prior to seeding of HEK293 cells.

### Creation of strain 576mn and complementation

Strain creation was essentially as previously described [9], with minor differences. The 2253 bp ∆*purM*::*FRT2-ble-FRT2* fragment from pGEM T-Easy ∆*purM*::*FRT2-ble-FRT2* (pPS2336; [9]) was removed by EcoRI digest and cloned into EcoRI digested pExKm5 [49, 50] by blue-white selection in NEB5a cells and verified by Zeo^R^ and enzyme digest. 200 ng of the resulting plasmid, pExKm5-∆*purM*::*FRT2-ble-FRT2*, was electroporated into electrocompetent *Bp* 576a prepared as previously described [53]. Merodiploids were selected on LB + Zeo containing media with X-gluc and resolved by sucrose counter-selection on LB + Zeo + sucrose containing media. Mutants were patched for verification of adenine auxotrophy and then PCR verified using oligos 1487 (5′-CACACGTAGAACGTGCGATC) and 1585 (5′-CTTTCGAGAAGCTTTCGACGG) purchased from Integrated DNA Technologies, Coralville, IA. An increase in size due to *FRT2-ble-FRT2* insertion was observed. Auxotrophy was verified by patching onto M9 glucose media +/− thiamine and adenine. Flp-excision of the *FRT2-ble-FRT2* cassette was accomplished by electroporating the pFlpe4 plasmid [49, 50] and selection on LB + Km. Colonies were streaked on LB + Km + rhamnose to induce Flp expression at room temperature. Colonies were patched on LB and LB + Zeo and incubated at 42 °C to cure plasmid and verify Zeo^S^. Zeo^S^ isolates were further confirmed to be Km^S^ and ade^−^ by patching on media lacking adenine and LB + Km. PCR confirmation of these strains showed a reduction in size across the *FRT2* lesion to just below wild-type. Complementation of the *Bp* 576a ∆*purM*::*FRT2* (named 576mn) was accomplished by inserting *purM* in single copy into the chromosome using the previously described mini-Tn*7* system single-copy complementation method [49, 52, 54]. Km^R^ transformants were verified by PCR and restoration of auxotrophy.

### LPS isolation

A modified hot-phenol extraction was utilized to extract LPS from select agent excluded and select agent *Bp*. This was done essentially as described [55] but with modifications included for BSL-3 activities. Each bacterial strain was grown on 8–10 plates of TSA or LB-agar for 48–72 h. Bacterial lawns were flooded with TBS and scraped off using a plate spreader. The bacterial suspensions were aliquoted into 2 mL O-ring gasketed microcentrifuge tubes and heat-killed at 110 °C for 15 min. Phenol was added to the lysed solution to a final concentration of 50% and 10% of the resulting mixture was plated on TSA to ensure sterility. Upon verification of sterility, the samples were moved to the BSL-2 laboratory and the protocol was continued as described. Samples were dialyzed using tubing with 12–14 kDa molecular weight cutoff against distilled water for 3–5 days until free of phenol. Samples from both phenol and aqueous phases were checked for presence of LPS by silver staining with 1026b LPS partitioning to the phenol phase and 576a partitioning to the aqueous phase while LPS. The phases of each LPS isolation were combined, lyophilized, treated with DNase I for 2 h, RNase H for 2 h, and Proteinase K overnight, then further purified as previously described [55]. To further purify, samples were lyophilized and resuspended in 50 mM ammonium acetate. 10 mg samples of LPS were FPLC purified using size exclusion chromatography with a HiPrep Sephacryl S-300 high-resolution column. An Agilent refractive index detector (RID), in line with an AKTA Purifier liquid chromatography system, was used to analyze and fractionate highly pure samples.

### SDS-polyacrylamide gels and western blots

Purified LPS (10 μg each) from wild-type strains 1026b and 576a and an equal amount of heat-killed lysate from Bp82 and 576mn were separated on SDS-polyacrylamide gels with a 12% resolving gel and a 4% stacking gel. Silver stains were carried out with the Pierce Silver Stain Kit (Thermo Scientific). Coomassie stains were performed using established methods. Colorimetric Western blots were performed by semi-dry electroblotting of SDS-PAGE run gels onto methanol soaked Immobilon P^SQ^ PVDF membranes from Millipore™ or Odyssey nitrocellulose membranes from LI-COR™. Blots were washed with 1xPBS, blocked with 1% skim milk in PBS and detected with 1-Step Ultra TMB-Blotting Solution (Thermo Scientific™) following standard practices and manufacturer’s instructions. Type A LPS mAb 4C7-HRP and type B mAb 5B4-HRP were kindly provided by Dr. David AuCoin and as previously described [24]. Rhesus macaque serum from a 1026b aerosol challenged monkey at 28 days post challenge was provided by Battelle, OH and was used at a 1:1000 dilution. Macaque antibodies were detected with anti-monkey IgA, IgG, IgM (H + L)-HRP (Sigma) secondary.

### Non-human primate LPS ELISA

Non-human primate serum isolated from rhesus macaques that had been aerosol challenged with 4 different type A LPS strains (1026b, K96243, HBPUB10303a, and HBPUB10134a) at different time points in relation to challenge were generously provided by Battelle. Immulon 4HBX flat bottom plates were coated with 1 μg/mL of the purified LPS in PBS at room temperature overnight. The plates were washed three times with PBS/T buffer, blocked with 5% skim milk in PBS/T for 1 h and washed three times with PBS/T buffer again. Serum samples were diluted 1:500 in blocking solution and incubated in the plates for 1 h followed by washing. Detection was carried out by incubating for 1 h with anti-Monkey IgG (γ-chain specific)-conjugated to peroxidase (Sigma) diluted 1:1000 in blocking solution. After 1 h, wells were washed 3 times then detected with Ultra-TMB ELISA solution. Reaction was stopped by addition of 1 M H_3_PO_4_. Absorbance was measured at 450 nm.

### Cell attachment assays

The attachment assay was performed by diluting *Bp* strains grown in LB medium overnight at 37 °C in PBS to an MOI of 1:1 in Dulbecco’s Modified Eagle Medium (DMEM). The dilutions were used to infect A549 human lung epithelial cells or RAW264.7 macrophages in 96-well CellBIND plates (Corning) at an MOI of 1:1. At 1 h post infection the bacteria-containing medium was removed and the monolayers were washed 3 times with pre-warmed PBS. Monolayers were lysed with 0.2% Triton-X100 in PBS, diluted, plated onto LB agar plates and incubated at 37 °C for 48 h. Colonies were enumerated and attachment efficiency was determined by dividing the attached number by the initial number of infecting bacteria, as determined by dilution plating on LB agar. The experiment was carried out in triplicate and the numbers represent the average of all three replicates with the error bars representing the SEM. The student *t*-test was used to determine the significance between attachment efficiencies of the wildtype strains.

### Plaque assays

Plaque assays were carried out essentially as previously described [56–58]. *Bp* strains 1026b, 576a, and MSH840 were grown overnight in LB medium at 37 °C, diluted and used to infect A549 and RAW264.7 monolayers at an MOI of 1:1 in 24-well CellBIND plates. After 1 h of infection the bacteria containing media was washed off the monolayers and further washed once more with PBS. Then 1.2% low-melt agarose (Fisher) in DMEM was heated to 60 °C, cooled to ~37 °C, then amikacin and kanamycin at 1000 μg/ml each were added. 500 μl was used to overlay each monolayer and they were incubated for 24 h at 37 °C in 5% CO_2_. The monolayers were fixed with 4% paraformaldehyde (PFA) in PBS for 45 min and the agarose plugs were removed. Monolayers were then stained with a 1% crystal violet solution and washed twice with deionized water for ease of viewing. Pictures of the monolayers were transformed into black and white images, inverted, and analyzed with ImageJ software (National Institutes of Health) to determine plaque number and diameter. All plaques were analyzed. Numbers presented are the average with the SEM.

### Invasion, intracellular replication and cytotoxicity assay

Intracellular replication assays were carried out as previously described [8, 35, 52]. Briefly, HEK293 cells infected with 1026b, 576a, Bp82, 576mn, and 576mn COMP in triplicate at an MOI of 1:1 (as determined by plating dilutions of the initial inocula) in an aminoglycoside protection assay. Bacteria were incubated with cells for 1 h then the monolayers were washed with 1xPBS three times. Amikacin and kanamycin each at 1000 μg /ml in DMEM were added to kill extracellular bacteria and inhibit extracellular growth for the remainder of the experiment. The *T* = 2 h time point was 1 h after the addition of the antibiotics. Monolayers were washed 3 times with 1xPBS and lysed with 0.2% Triton-X100 in PBS at 2, 24, and 48 h post-infection. Undiluted and diluted lysates were plated and bacterial CFU enumerated. Cytotoxicity assays were carried out as above in triplicate but cell media supernatant was removed and the Pierce LDH Cytotoxicity Assay kit was used to measure LDH release in the media by following the manufacturers recommendations. Sample levels were compared to total cell lysis and spontaneous lysis controls from the 48 h time point to obtain % cytotoxicity.

### Wild-type *B. pseudomallei* pre-determined endpoint and mutant attenuation animal studies

All Select Agent animal work was carried out in a CDC/USDA Tier 1 approved facility at the University of Florida following Tier 1 regulations. All protocols were approved by the Institutional Animal Care and Use Committee at the University of Florida prior to implementation. Female BALB/c mice between 4 and 6 weeks of age were purchased from Jackson Laboratories (Bar Harbor, ME). Animals were housed in microisolator cages under pathogen-free conditions. Strains were grown overnight and frozen in 20% glycerol aliquots overnight at −80 °C. An aliquot of each was thawed and CFU enumerated by dilution plating LB + ade medium. Dilution values were determined for the target inoculation CFU of 5000 CFU in 20 μl of PBS, or 1 × 10^6^ CFU in the case of the mutant strains. Animals were anesthetized with 100 mg/kg of ketamine (Patterson Veterinary) of body weight plus 10 mg/kg xylazine. Once fully anesthetized, groups of 5 mice (*n* = 5) were challenged with the 20 μl inoculum by pipetting into the nares of the mouse alternating nostrils until fully inhaled. For the pre-determined endpoint study, mice were humanely euthanized at 24 h. The lungs, liver, spleen, and an aliquot of blood were isolated and organs were processed in 5 ml of 1xPBS using a stomacher (Seward). Undiluted and diluted aliquots were plated on LB for CFU determination. Colonies were positively identified as *Bp* by testing with the latex agglutination test as previously described [59, 60]. Lung homogenate was mixed with Protease Inhibitor Cocktail (Sigma) and frozen at −80 °C for TNF-α measurements. Virulence studies were carried out exactly as above but using wild-type and mutant *Bp* strains. Mice were observed twice daily for the first 4 days then once daily until the end of the 14 d study. Mice were euthanized at humane endpoints or when moribund. Mice that survived until the end of the study had the organs removed and processed for bacterial loads as described above but using LB + ade.

### TNF-α detection in lung homogenates

Lung homogenates were filtered through sterile Costar Spin-X centrifuge tube filters with cellulose acetate membranes of pore size 0.22 μm (Corning). All samples were checked for sterility after 48 h growth on LB + adenine. TNF-α levels were measured per organ determined by comparison to a standard curve using the Mouse TNF-α Quantikine ELISA Kit (R&D Systems).

### Statistics

Statistical analysis was carried out using the GraphPad Prism version 6 software. Significant differences between 1026b and 576a attachment efficiencies and plaque sizes were determined by unpaired t test assuming a normal distribution with the standard error of the mean. Organ load differences were subjected to the Mann Whitney test of the medians due to the number of data points in each group. TNF-α levels in lung homogenates were compared using the ordinary one-way ANOVA to compare the three groups. All survival curves were compared using the log-rank (mantel-Cox) test for significance. Ordinary one-way ANOVA was used to compare invasion efficiencies and cytotoxicity % amongst multiple samples. To compare multiple time points and multiple samples from the intracellular replication experiment a two-way ANOVA was utilized.

## Additional files

Additional file 1: NHP LPS ELISA using rhesus macaque serum samples (n=) isolated at the indicated day post aerosol challenge with strains possessing type A LPS. Plates were coated with pure type A LPS (black bars) or type B LPS (white bars) and the values shown are the average of the IgG serum reactivity (n) at each day. Error bars represent the SEM. (PDF 37 kb)

Additional file 2: HEK293 Invasion Assay. All strains invaded HEK293 cells equally well. Significance was tested by one-way ANOVA. (PDF 4607 kb)

## Abbreviations

ABSL3: Animal biosafety level 3
Ade: Adenine
*Ble*: Bleomycin resistance gene
*Bp*: *Burkholderia pseudomallei*
BSL2: Biosafety level 2
BSL3: Biosafety level 3
CDC/USDA: Centers for Disease Control and Prevention/United States Department of Agriculture
CFU: Colony forming unit
COMP: complement
DMEM: Dulbecco’s modified eagles medium
FPLC: Fast performance liquid chromatography
*FRT*: Flp recognition target
HRP: Horse radish peroxidase
LB: Lysogeny broth
LD_50_: Median lethal dose
LPS: Lipopolysaccharide
MOI: Multiplicity of infection
PBS: Phosphate buffered saline
PBS/T: Phosphate buffered saline with tween
*purM*: Phosphoribosyl-aminoimidazole (AIR) synthase
SDS-PAGE: Sodium dodecyl sulfate-polyacrylamide gel electrophoresis
TNF-α: Tumor necrosis factor alpha
TSA: Tryptic soy agar
*wcbB*: Mannosyltransferase essential for *Bp* capsule synthesis
zeo: Zeocin
