# Supplementary figures and images for: An avirulent Burkholderia pseudomallei ∆purM strain with atypical type B LPS: expansion of the toolkit for biosafe studies of melioidosis
Source: BMC Microbiol. 2017 Jun 7;17:132. doi: 10.1186/s12866-017-1040-4 (PMC5461690; doi:10.1186/s12866-017-1040-4)

# NHP LPS ELISA

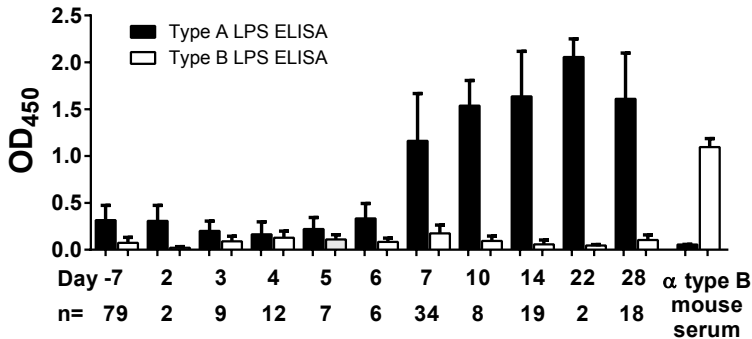

Supplement: Supplementary file 1 — NHP LPS ELISA using rhesus macaque serum samples (n=) isolated at the indicated day post aerosol challenge with strains possessing type A LPS. Plates were coated with pure type A LPS (black bars) or type B LPS (white bars) and the values shown are the average of the IgG serum reactivity (n) at each day. Error bars represent the SEM. (PDF 37 kb) [file 12866_2017_1040_MOESM1_ESM.pdf]
